# Electrical impedance myography as a biomarker of myostatin inhibition with ActRIIB-mFc: a study in wild-type mice

**DOI:** 10.4155/fsoa-2018-0002

**Published:** 2018-04-16

**Authors:** Janice A Nagy, Kush Kapur, Rebecca S Taylor, Benjamin Sanchez, Seward B Rutkove

**Affiliations:** 1 Department of Neurology, Beth Israel Deaconess Medical Center, 330 Brookline Ave, Boston, MA 02215, USA; 2 Department of Neurology, Boston Children's Hospital, Harvard Medical School, 300 Longwood Avenue, Boston, MA 02115, USA

**Keywords:** ActRIIB-mFc, electrical impedance, mouse, myofiber hypertrophy, myography, myostatin inhibition, phase, reactance, resistance

## Abstract

**Aim:**

We sought to determine the sensitivity of electrical impedance myography (EIM) to myofiber hypertrophy induced by treatment with various doses of ActRIIB-mFc, an inhibitor of myostatin signaling.

**Methods:**

Wild-type C57BL/6 J mice (n = 40, male) were treated with three different doses of ActRIIB-mFc (i.e., RAP-031) or vehicle twice weekly for 5 weeks. End point assessments included gastrocnemius EIM, force measurements, muscle mass and myofiber size quantification.

**Results:**

ActRIIB-mFc increased body mass, muscle mass and myofiber size across all doses. Alterations in EIM 50 kHz phase and center frequency (*fc*) were also present, with trends in a dose-dependent fashion. Significant correlations between EIM parameters and myofiber/functional data were identified.

**Conclusion:**

EIM outcomes can serve as effective biomarkers of myostatin signaling inhibition, demonstrating a dose sensitivity and correlation to standard assessments.

## Lay abstract

We were interested in studying the sensitivity of a technique, called electrical impedance myography (EIM), to noninvasively assess the size of muscle fibers. In this technique a minute electrical current is used to probe the tissue. To do so, we gave a drug (ActRIIB-mFc) to mice that enlarges muscle fibers at three different doses. We were able to show that the EIM technique was able to detect this differential effect and functional changes induced by the drug correlated to the EIM data. This work suggests that EIM will be useful as a noninvasive marker muscle health.

Better biomarkers to assess drug effect in neuromuscular disease clinical trials are needed. Although standard clinical outcome measures, such as functional testing, handheld dynamometry [1] and the 6-min walk test [2], are valuable, they also have a variety of drawbacks. These include most importantly requiring the cooperation and understanding of the patient undergoing testing and a skilled physical therapist or evaluator performing the measurements. Yet, these outcomes can still be affected by the emotional state of the patient and can be fatiguing and time consuming to perform. The use of such measures often translates into large sample size requirements and long study periods in order to identify treatment effects for many of these disorders [3].

For this reason, there has been a strong effort to establish pharmacodynamic biomarkers for the assessment of neuromuscular disease; that is, markers that are sensitive to drug efficacy. Electrical impedance myography (EIM) is an impedance-based technique that has been receiving increasing attention as a tool that could serve such a role in clinical trials [4]. Multiple studies have demonstrated its sensitivity to disease progression, including in amyotrophic lateral sclerosis [5], Duchenne muscular dystrophy [6], spinal muscular atrophy [7] and even potentially in sarcopenia [8,9]. However, there has been relatively little work to date assessing its potential sensitivity to drug effect and there has been no study to our knowledge assessing its ability to detect a dose–response relationship.

In this study, we sought to assess that final point by performing a basic assessment of EIM's sensitivity to dose by measuring a group of wild-type mice treated with three different doses of ActRIIB-mFc, and its relationship to myofiber size and function. ActRIIB-mFc (also termed RAP-031, Acceleron Pharma, MA, USA) is a protein comprised of a form of the extracellular domain of ActRIIB fused to a mouse Fc that acts as a ligand trap to inhibit myostatin signaling [10,11]. Previously, only one other study evaluated EIM's response to ActRIIB-mFc, and that was only at a single full dose [12]. Administration of either ActRIIB-mFc or ActRIIB-hFc has been shown to result in a substantial muscle mass increase in normal mice [11–18], and thus in this study we sought to evaluate EIM's sensitivity to dose, helping to further establish its potential value as biomarker of muscle status.

## Materials & methods

### Animals

All animal procedures were carried out in strict accordance with the recommendations in the *Guide for the Care and Use of Laboratory Animals* of the NIH and approved by the Institutional Animal Care and Use Committee at Beth Israel Deaconess Medical Center (Protocol #087–2016). Forty male wild-type mice (C57BL6J, 8 weeks of age) were obtained from the Jackson Laboratories (ME, USA). Mice were given *ad libitum* access to food (Formulab Diet 5008, LabDiet, MO, USA) and water. Animals were allowed to acclimate at least 72 h prior to testing. At the conclusion of all studies, the animals were euthanized with carbon dioxide.

### Treatment with the myostatin ligand trap ActRIIB-mFc

Starting at 9 weeks of age, mice were divided randomly into four groups of 10 mice per group. Mice were treated twice weekly for 5 weeks with subcutaneous (sc.) injections of either phosphate-buffered saline (PBS) or ActRIIB-mFc at doses of 3.3, 6.6 or 9.9 mg/kg. These doses were based on previous literature [10–12]. Animals were weighed weekly with an analytical balance to ensure correct dosing throughout the course of the study. All subsequent procedures, described below, were performed after 5 weeks of treatment with ActRIIB-mFc.

### Grip strength assessment

Fore limb and hind limb grip strength were measured by a grip strength meter with standard pull bar (CAT #1027CSM, Columbus Instruments, OH, USA). The mouse was first allowed to grip the pull bar and then the investigator, grasping the lower back of the animal, pulled the animal away from the bar until it lost its grip. The maximum force out of five trials was recorded. This test was performed separately on the fore and hind limbs.

### Animal preparation for EIM & *in situ* force measurements

EIM and *in situ* force experiments were performed under 1–2% inhaled isoflurane anesthesia delivered by nose cone with body and muscle temperature being maintained by a heating pad (37 °C). Both hind limbs were taped to the measuring surface at an approximately 45° angle extending out from the body in preparation for measurements.

### Electrical impedance myography

After shaving the left hind limb, a depilatory agent was then applied to remove residual fur, and the skin was cleaned with 0.9% saline solution. A fixed rigid four-electrode impedance-measuring array was applied over the left gastrocnemius muscle. EIM measurements were performed with the mView EIM System (Myolex, Inc., CA, USA), obtaining impedance data at 41 frequencies from 1 kHz to 10 MHz as previously described [19]. The device was connected to the electrode array via a ‘cradle’ adapter available from the company. The two major outcomes of interest in this study were the 50 kHz phase, extracted from the multifrequency data and the ‘center frequency’ (*fc*), a multifrequency measure thought to represent the relative size of cells [20].

### *In situ* muscle force measurements

*In situ* muscle force measurements were performed, as previously described [12]. Briefly, a nonsurvival surgery was performed to expose the left gastrocnemius muscle and calcaneal tendon, the latter of which was cut and attached via silk suture to the force lever arm and the leg stabilized by inserting a disposable monopolar needle (902-DMF37-S, Natus Neurology, WI, USA) through the knee joint. Twitch and tetanic force were recorded following stimulation of the sciatic nerve with 200 ms square pulses via insulated monopolar needles (F-E2M-48, Grass Technologies, RI, USA). A high-speed servo motor-based apparatus (Model 305C, Aurora Scientific, ON, Canada) was used to measure force output. Stimulation current and resting tension were adjusted to maximize twitch force produced by a single stimulus pulse. All subsequent isometric tetanic force data were collected at this stimulation current and resting tension. Isometric force frequency relationship was recorded after stimulation by a train of square wave stimuli and maximum isometric tetanic force was recorded. Animals were then euthanized.

### Muscle histology

After euthanasia, the left gastrocnemius was harvested and the wet mass of the excised muscle was determined using a standard analytical balance. Normalized muscle mass was calculated by taking this wet muscle mass and dividing by the mass of the mouse prior to muscle excision. Muscle tissue was placed in 10% buffered formalin and fixed for at least 48 h. Samples were then embedded in paraffin blocks, sectioned into 10 mm slices and stained with anticollagen VI antibody (Abcam ab6588). Sections were subsequently imaged at 20× with a Zeiss Axio Imager M1 epifluorescence microscope and fiber area was measured using Volocity^®^ software (PerkinElmer, Akron, OH, USA).

### Data analysis

The EIM resistance (R) and reactance (X) were extracted at 50 kHz and phase (θ) was calculated via the equation θ = arctan (X/R). Multifrequency resistance and reactance data were then used to extract the Cole parameters, a series of parameters that offer potential information on tissue histology: these include α (a measure of cell size variation), R0/Rinf (a measure of cell density) and *fc* (a myofiber size) [12,20,21]. Statistical analysis on the physiological data, the raw impedance values and the Cole parameters was then performed using GraphPad Prism (GraphPad Software, Inc., CA, USA). All mass, grip strength, force and 50 kHz EIM data are reported as mean ± standard error of the mean. Multiple group comparisons were performed by ordinary one-way analysis of variance (ANOVA) with *post-hoc* t-tests using Tukey's multiple comparisons test. For correlation analyses, the Pearson correlation coefficient was calculated. Findings were considered significant with p < 0.05, two tailed.

## Results

### Basic data

**Figure 1.**
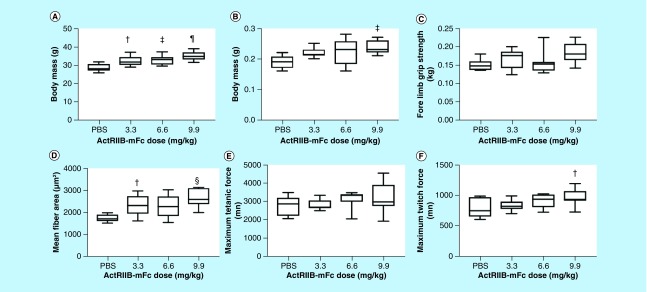
**Summary of basic physiological parameters and force data across all three doses of ActRIIB-mFc.** **(A)** Body mass; **(B)** muscle mass; **(C)** fore limb grip strength; **(D)** mean fiber area; **(E)** gastrocnemius *in situ* tetanic force; **(F)** gastrocnemius *in situ* twitch force. ^†^p < 0.05; ^‡^p < 0.01; ^§^p < 0.001; ^¶^p < 0.0001. *Post-hoc* p-values are reported for the comparison between each individual ActRIIB-mFc dose versus the phosphate-buffered saline control. *Post-hoc* p-values for the comparison between the various ActRIIB-mFc doses were not significant. ns: Not significant.

Figure 1 shows the most interesting standard outcomes after 5 weeks of treatment on the standard metrics of body mass, muscle mass, fore limb grip strength, mean fiber area, maximum tetanic force and maximum twitch force. The complete dataset and associated statistics are also provided in Table 1. Trends are apparent in almost all parameters with increasing/improving parameters paralleling increasing ActRIIB-mFc dose. Significant ANOVA were observed with body mass, muscle mass, fore limb grip strength, mean fiber area and maximum twitch force with *post-hoc* tests confirming a difference only with PBS compared with each of the doses (and not between the different doses). This finding suggests that our choice of dosing may have been less than ideal, since there appeared to be, in general, relatively minor difference between effects of 3.3, 6.6 and 9.9 mg/kg.

**Table 1.** **Body mass, muscle mass, grip strength, muscle force and myofiber size.**

**Characteristic**	**PBS (n = 9)**	**ActRIIB-mFc dose mg/kg**			**ANOVA p-value**	***Post-hoc* p-value**

		**3.3 (n = 10)**	**6.6 (n = 9)**	**9.9 (n = 10)**		
Body mass (g)	28.06 ± 0.75	32.17 ± 0.77	32.74 ± 0.81	34.61 ± 0.76	0.0001	0.0035 0.0011 <0.0001

Absolute muscle mass (g)	0.191 ± 0.0070	0.220 ± 0.0055	0.224 ± 0.013	0.234 ± 0.0075	0.0065	ns ns 0.0059

Normalized muscle mass (g/g)	0.0069 ± 0.0003	0.0069 ± 0.0002	0.0068 ± 0.0003	0.0068 ± 0.0002	ns	ns ns ns

Fore limb grip strength (g)	146.6 ± 5.5	166.6 ± 7.8	159.6 ± 10.1	181.6 ± 8.0	0.0314	ns ns 0.0201

Hind limb grip strength (g)	34.0 ± 5.2	37.9 ± 9.2	32.9 ± 5.6	47.7 ± 6.5	ns	ns ns ns

Normalized fore limb grip strength (g/g)	5.234 ± 0.178	5.177 ± 0.215	4.883 ± 0.300	5.263 ± 0.239	ns	ns ns ns

Normalized hind limb grip strength (g/g)	1.235 ± 0.213	1.167 ± 0.266	1.004 ± 0.170	1.378 ± 0.194	ns	ns ns ns

Twitch force (mN)	767.2 ± 50.02	829.6 ± 33.97	905.4 ± 34.83	939.9 ± 42.35	0.0233	ns ns 0.0246

Tetanic force (mN)	2783 ± 163	2871 ± 123	3145 ± 153	3167 ± 238	ns	ns ns ns

Normalized twitch force (mN/g)	27.38 ± 1.67	25.94 ± 1.31	27.82 ± 1.36	27.29 ± 1.34	ns	ns ns ns

Normalized tetanic force (mN/g)	99.65 ± 6.18	89.80 ± 4.58	96.13 ± 4.62	92.15 ± 7.21	ns	ns ns ns

Mean fiber area (μm^2^)	1673 ± 65	2310 ± 131	2284 ± 167	2621 ± 124	0.001	0.0062 0.0114 <0.0001

For muscle fiber cross-sectional area: PBS (1680 fibers); ActRIIB-mFc @ 3.3 mg/kg (1486 fibers); ActRIIB-mFc @ 6.6 mg/kg (1299 fibers); ActRIIB-mFc @ 9.9 mg/kg (1255 fibers) were analyzed. Grip strength and muscle force were normalized to body mass. Data shown as mean ± standard error of the mean.

*Post-hoc* p-values are reported for the comparison between each individual ActRIIB-mFc dose versus the PBS control. None of the *post-hoc* p-values for the comparison between the various ActRIIB-mFc doses were significant.

ANOVA: Analysis of variance; ns: Not significant; PBS: Phosphate-buffered saline.

### EIM & Cole parameters

**Figure 2.**
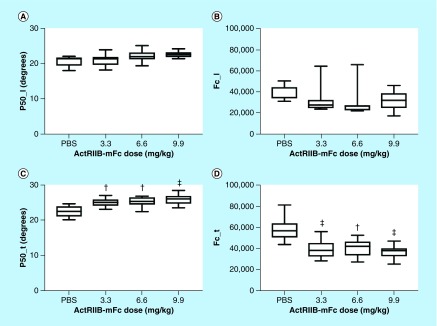
**Longitudinal and transverse electrical impedance myography and Cole parameters for the left gastrocnemius muscle.** **(A)** Longitudinal 50 kHz phase; **(B)** longitudinal *fc*; **(C)** transverse 50 kHz phase; **(D)** transverse *fc*. ^†^p < 0.05; ^‡^p < 0.01. *Post-hoc* p-values are reported for the comparison between each individual ActRIIB-mFc dose versus the phosphate-buffered saline control. *Post-hoc* p-values for the comparison between the various ActRIIB-mFc doses were not significant. *fc*: center frequency; ns: Not significant.

Both the EIM transverse 50 kHz phase and the Cole *fc* parameter were highly significantly different in all three ActRIIB-mFc-treated groups as compared with the animals treated with PBS (Figure 2 & Table 2); the data in the longitudinal direction were not significantly different from that found in PBS-treated animals for mice treated with any of the ActRIIB-mFc doses.

**Table 2.** **Longitudinal and transverse 50 kHz electrical impedance myography and Cole parameters of the left gastrocnemius.**

**Direction of measurement**	**Impedance parameter**	**PBS (n = 8)**	**ActRIIB-mFc dose mg/kg**			**ANOVA p-values**	***Post-hoc* p-value**

			**3.3 (n = 8)**	**6.6 (n = 8)**	**9.9 (n = 9)**		
Longitudinal	50 kHz phase	20.77 ± 0.525	21.04 ± 0.619	22.18 ± 0.601	22.63 ± 0.295	0.0451	ns ns ns

	50 kHz reactance	90.02 ± 3.49	80.66 ± 6.01	92.41 ± 4.72	89.00 ± 2.26	ns	ns ns ns

	50 kHz resistance	237.0 ± 4.98	207.7 ± 10.82	225.9 ± 7.29	213.4 ± 3.78	0.0317	0.0347 ns ns

Transverse	50 kHz phase	22.61 ± 0.567	25.13 ± 0.453	25.34 ± 0.499	25.97 ± 0.507	0.0004	0.0088 0.0043 0.0003

	50 kHz reactance	126.6 ± 6.50	135.5 ± 7.51	155.6 ± 12.35	134.5 ± 4.99	ns	ns ns ns

	50 kHz resistance	304.6 ± 15.14	288.5 ± 13.81	332.8 ± 34.94	275.5 ± 5.73	ns	ns ns ns

Longitudinal	*fc* (kHz)	37,705 ± 2475	31,873 ± 4760	30,127 ± 5150	31,482 ± 3219	ns	ns ns ns

	R0/Rinf	3.67 ± 0.206	3.68 ± 0.298	3.846 ± 0.229	4.34 ± 0.087	ns	ns ns ns

	α	0.7506 ± 0.0228	0.7093 ± 0.0116	0.7602 ± 0.0208	0.7075 ± 0.0082	ns	ns ns ns

Transverse	*fc* (kHz)	58,423 ± 4021	38,875 ± 3182	40,097 ± 2940	36,487 ± 2125	<0.0001	0.0007 0.0015 0.0001

	R0/Rinf	5.414 ± 0.344	6.065 ± 0.337	5.861 ± 0.346	6.385 ± 0.236	ns	ns ns ns

	α	0.7347 ± 0.0243	0.7131 ± 0.010	0.7481 ± 0.0254	0.7145 ± 0.0095	ns	ns ns ns

Phase shown in degrees, and reactance and resistance are shown in ohms. Data presented as mean ± standard error of the mean.

*Post-hoc* p-values are reported for the comparison between each individual ActRIIB-mFc dose versus the PBS control. None of the *post-hoc* p-values for the comparison between the various ActRIIB-mFc doses were significant.

ANOVA: analysis of variance; *fc*: Center frequency; ns: Not significant; PBS: Phosphate-buffered saline.

### Correlation to myofiber size & impedance parameters

**Figure 3.**
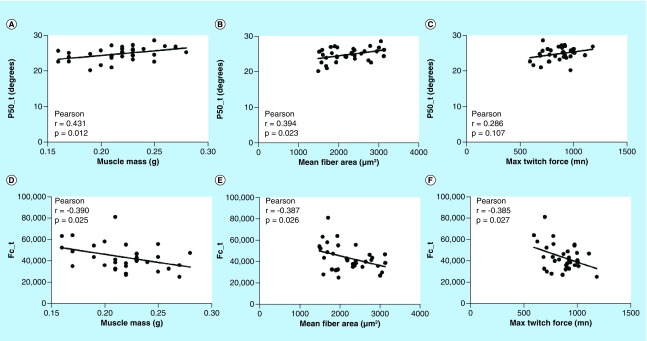
**Correlations between transverse electrical impedance myography and Cole parameters, and muscle mass, mean fiber area and maximum twitch force.** **(A)** Transverse 50 kHz phase versus muscle mass; **(B)** transverse 50 kHz phase versus mean fiber area; **(C)** transverse 50 kHz phase versus maximum twitch force; **(D)** transverse *fc* versus muscle mass; **(E)** transverse *fc* versus mean fiber area; **(F)** transverse *fc* versus maximum twitch force. *fc*: Center frequency.

Figure 3 shows the correlation between two impedance parameters (i.e., p50_t and *fc*_t) with muscle mass, myofiber size and maximum twitch force across all the animals at all doses demonstrating significant relationships across all groups, except for the p50_t versus twitch force. The full dataset is provided in Table 3.

**Table 3.** **Correlations between 50 kHz electrical impedance myography parameters, grip strength, muscle mass, twitch force, tetanic force & mean cross-sectional area.**

**Impedance feature category**	**Specific impedance value**	**Body mass**	**Muscle mass**	**Fore limb grip strength**	**Maximum twitch force**	**Maximum tetanic force**	**Mean fiber area**
Longitudinal 50 kHz EIM parameters	Phase	r = 0.261 p = 0.142	r = 0.247 p = 0.166	r = 0.222 p = 0.215	r = 0.210 p = 0.241	r = 0.219 p = 0.220	r = 0.041 p = 0.822

	Reactance	r = -0.164 p = 0.363	r = -0.134 p = 0.456	r = -0.197 p = 0.272	r = 0.047 p = 0.797	r = 0.033 p = 0.857	r = -0.268 p = 0.132

	Resistance	r = -0.455 p = 0.0078	r = -0.400 p = 0.021	r = -0.465 p = 0.006	r = -0.091 p = 0.614	r = -0.112 p = 0.534	r = -0.408 p = 0.018

Longitudinal Cole parameters	*fc*	r = -0.192 p = 0.284	r = -0.123 p = 0.494	r = -0.169 p = 0.347	r = -0.174 p = 0.332	r = -0.115 p = 0.524	r = -0.088 p = 0.627

	R0/Rinf	r = 0.200 p = 0.264	r = 0.156 p = 0.385	r = 0.171 p = 0.340	r = 0.172 p = 0.339	r = 0.100 p = 0.581	r = 0.111 p = 0.539

	α	r = -0.366 p = 0.0361	r = -0.180 p = 0.317	r = -0.200 p = 0.265	r = -0.048 p = 0.790	r = 0.013 p = 0.942	r = -0.320 p = 0.069

Transverse 50 kHz EIM parameters	Phase	r = 0.482 p = 0.0045	r = 0.431 p = 0.012	r = 0.203 p = 0.258	r = 0.286 p = 0.107	r = 0.116 p = 0.521	r = 0.394 p = 0.023

	Reactance	r = 0.235 p = 0.188	r = 0.234 p = 0.154	r = -0.263 p = 0.139	r = 0.058 p = 0.750	r = 0.115 p = 0.526	r = 0.231 p = 0.196

	Resistance	r = 0.018 p = 0.923	r = 0.064 p = 0.723	r = -0.326 p = 0.064	r = -0.080 p = 0.659	r = 0.063 p = 0.727	r = 0.046 p = 0.800

Transverse Cole parameters	*fc*	r = -0.495 p = 0.0034	r = -0.390 p = 0.025	r = -0.280 p = 0.115	r = -0.385 p = 0.027	r = -0.218 p = 0.223	r = -0.386 p = 0.026

	R0/Rinf	r = 0.253 p = 0.156	r = 0.543 p = 0.001	r = 0.177 p = 0.325	r = 0.438 p = 0.011	r = 0.376 p = 0.031	r = 0.169 p = 0.346

	α	r = -0.083 p = 0.647	r = -0.348 p = 0.047	r = -0.284 p = 0.110	r = -0.457 p = 0.007	r = -0.465 p = 0.006	r = -0.054 p = 0.764

Data from a total of 33 mice (8 PBS; 8 ActRIIB-mFc @ 3.3 mg/kg; 8 ActRIIB-mFc @ 6.6 mg/kg; 9 ActRIIB-mFc @ 9.9 mg/kg) were included for each correlation. Pearson correlation coefficients (r) and p values (p) are shown.

EIM: Electrical impedance myography; *fc*: Center frequency; PBS: Phosphate-buffered saline.

## Discussion

Taken together, these results confirm the capability of EIM to detect a relationship to myofiber size and other outcomes with varying doses of ActRIIB-mFc, a potent inducer of myofiber hypertrophy. The greatest limitation of our study design was our choice of doses to test; whereas we anticipated a linear impact on myofiber size with escalating dose, in fact, no significant difference could be identified for any outcome assessment across the three doses, except for animal body mass and mean fiber area. Nevertheless, the trends across the three doses were apparent on histology, functional measures and the EIM data itself.

Other studies evaluating EIM's sensitivity to actual drug effect have been limited. There has been one previous study evaluating EIM previously detecting the impact of myostatin inhibition of ActRIIB-mFc (also termed RAP-031) [12]. But this study looked at relatively few animals, performed measurements in only a single direction (longitudinal), and studied only one dose. In addition, one human study identified the impact of corticosteroids on EIM in patients with Duchenne muscular dystrophy [6]. Perhaps the closest study that attempted to show a differential effect of medication on the EIM parameters was in spinal muscular atrophy mice, in which mice with a severe form of the disease (the SMNΔ7 mouse) were treated with identical doses of antisense oligonucleotides at different ages shortly after birth [22]; animals that were treated later had a more severe phenotype; those treated earlier had a milder phenotype. EIM was able to detect these differences.

In this study, we were mainly interested in a relatively restricted set of impedance parameters, namely the 50 kHz phase values and the Cole-modeled impedance parameter, *fc*, obtained both in the longitudinal and transverse directions. The main reason for not focusing on the raw 50 kHz resistance and reactance values in this situation is that the changes in these values more likely reflect changes in muscle mass rather than changes due to the inherent structure of the myofibers themselves. While there are several Cole parameters, *fc* is most closely tied to myofiber size and thus we anticipated the greatest changes in this one parameter.

The difference in transverse versus longitudinal data is also of interest. In transverse measurements, the electrical current is passed perpendicular to the fiber direction (i.e., current is passed in the medial–lateral direction and the voltage measured in that same direction); in longitudinal measurements, the current is passed parallel to the myofibers (i.e., proximal to distal). In the transverse direction, current is forced across myofibers and would thus be more likely to detect changes in their size than might be the case in the longitudinal direction; longitudinal changes would be anticipated in being more sensitive to changes in the interstitium.

There are several limitations to this study that need to be highlighted. The first, as already stated, was that the doses of ActRIIB-mFc we chose were too similar, all three inducing similar degrees of change in muscle fiber size. Perhaps, if we had still larger sample sizes, we may have been able to detect a significance difference between these doses, as trends do appear. Nevertheless, it would have been preferable to use a different and perhaps wider range of doses (e.g., 1, 2 and 5 mg/kg and possibly doses >10 mg/kg). Of note, a previous dose–response study indicated that the maximal efficacy on lean tissue mass was observed with the bi-weekly administration of ActRIIB-mFc at a dose 10 mg/kg in mice [17]. In addition, in this study we only evaluated wild-type animals, since our main goal was simply in demonstrating EIM's potential applicability as a biomarker of drug effect. A similar study in a disease in which we anticipate drug efficacy would be an important next step to pursue. Nevertheless, by studying the effect in wild-type animals, we provide the ‘cleanest’ example of EIM's potential capability of detecting a differential drug effect. A final more minor limitation is that we used formalin fixation in lieu of frozen, which may produce some degree of shrinkage in the tissue. However, such shrinkage is likely minor and would have affected all specimens similarly and thus not impact the overall results of the study. In addition, our untreated mouse myofiber size was well within the expected range for adult mice.

## Conclusion

The value of this study is that it demonstrates EIM's capability to offer data that are well correlated to standard measures that are more challenging to obtain. We show here that EIM parameters of 50 kHz phase and the *fc* show a moderate correlation to multiple features of the drug treatment, including the myofiber cross-sectional area, muscle mass and twitch/isometric force. Thus, it is conceivable that this technology may ultimately be able to serve as a biomarker or potentially as a surrogate outcome in future clinical trials. Given its ease of use, both in animals and in humans, it could serve as a simple measure of drug effect that provides direct correlation to histological and force-generating aspects of the muscle, without the need for invasive biopsy or extended functional measurements.

## Future perspective

We anticipate the broader neuromuscular disease field will gradually incorporate additional tools such as EIM to evaluate muscle condition in a variety of diseases rather than some of the standard functional outcome measures currently in use. The initial purpose will be to help speed clinical trials and therapeutic testing in general. In addition, it is likely that such approaches will begin to reach mainstream clinical care, especially as some of these tools can be used at home to provide a more detailed picture of therapeutic trends over time.

**Summary points**

- ActRIIB-mFc, a potent inducer of myofiber hypertrophy, increased body mass, muscle mass and myofiber size across all doses tested.
- Dose-dependent trends were observed in two electrical impedance myography (EIM) parameters: the 50 kHz phase and *fc*, the center frequency.
- Significant correlations were identified between EIM parameters and myofiber cross-sectional area.
- Significant correlations were identified between EIM parameters and muscle mass.
- Significant correlations were identified between EIM parameters and functional measures.
- EIM outcomes can serve as effective biomarkers of myofiber hypertrophy.
- EIM obviates the need for invasive biopsy or extended functional measurements and can function as a pharmacodynamic biomarker for the assessment of drug efficacy in neuromuscular disease.
